# LEDAW: An Integrated Software Suite with GUI for Automating Local Energy Decomposition Analysis of Molecular Interactions

**DOI:** 10.1021/acs.jcim.5c01561

**Published:** 2025-08-28

**Authors:** Ahmet Altun, Frank Neese, Giovanni Bistoni

**Affiliations:** † Max-Planck-Institut für Kohlenforschung, Kaiser-Wilhelm-Platz 1, D-45470 Mülheim an der Ruhr, Germany; ‡ Department of Chemistry, Biology and Biotechnology, University of Perugia, 06123 Perugia, Italy

## Abstract

Accurate interaction energies can be obtained using high-level quantum chemical methods such as DLPNO–CCSD­(T) and its HFLD variant in ORCA. The local energy decomposition (LED) scheme helps interpret these energies by breaking them down into chemically meaningful components. However, preparing LED inputs and analyzing results is often complex and error-prone. To streamline this process, we developed LEDAW (local energy decomposition analysis wizard), a Python-based tool that automates LED workflows. It supports standard and fragment-pairwise (fp)-LED, complete basis set (CBS) and Complete PNO Space (CPS) extrapolations, and analysis of N-body and cooperativity effects. With both a GUI and script-based workflow, LEDAW reduces analysis time from hours or days to just minutes, improving usability and reproducibility. It accelerates the generation of interaction energy matrices and heat maps, making advanced analysis of protein–ligand complexes, DNA assemblies, solute–solvent interactions, and molecular crystals more accessible.

## Graphical abstract

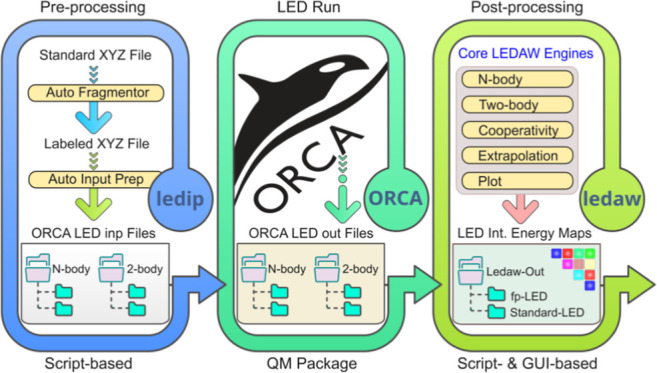

## 1 Introduction

The accurate quantification of chemical interactions is a cornerstone of modern chemistry and biology, underpinning phenomena ranging from molecular recognition in drug design and enzymatic catalysis to the assembly of complex materials and crystal structures. Among the plethora of quantum chemical methods developed for quantifying such interactions, the Coupled-Cluster Singles and Doubles with perturbative triples (CCSD­(T)) method is considered as the “gold standard” because its results often agree with experiments within their uncertainties.^1^ Its computational cost, however, scales steeply with system size, limiting practical applications to relatively small systems.

In contrast, its near-linear-scaling domain-based local pair natural orbital (DLPNO) variant, DLPNO–CCSD­(T), enables accurate treatment of large biomolecular systems and complex materials by exploiting the rapid decay of electron correlation with interelectronic distance.^2–6^ Further enhancing computational efficiency, the Hartree–Fock plus London Dispersion (HFLD) scheme introduces physically relevant assumptions for noncovalent interactions (NCIs).^7,8^ HFLD achieves accuracy intermediate between CCSD and CCSD­(T) on challenging benchmark sets,^7,8^ making it a reliable and efficient tool for large-scale systems such as proteins,^7^ DNA,^9^ solute–solvent interactions,^8^ and molecular crystals.^10–12^

The local energy decomposition (LED) scheme facilitates the interpretation of the computed energies by these methods, breaking them down into several physically meaningful terms, such as electrostatic, exchange, and London dispersion (LD) contributions. It enables multifragment analyses of both closed-shell^13^ and open-shell^14^ systems across both weak and strong interaction regimes. Its widespread application in benchmark studies,^15–17^ solute–solvent interactions,^8,18,19^ molecular clusters/crystals,^10–12,20^ biomolecular assemblies,^9^ protein–ligand interactions,^21–23^ catalysis,^24–26^ coordination chemistry,^27–30^ and spin-state energetics^14,31^ has profoundly enhanced the understanding of complex molecular interactions.^32–36^

The original LED scheme^13,14^ has undergone continuous refinement and extension to provide deeper insights into chemical interactions. Recent developments include estimation of triples contribution to LD,^29,36^ decomposition of LD into atomic contributions,^37^ distribution of the solute–solvent terms from implicit solvation schemes,^36^ fragment pairwise (fp)-LED scheme^36^ that extracts interaction strength between all fragment pairs in multifragment systems, and the COVALED scheme (available in ORCA 6.1+)^38–42^ allowing fragment definitions through covalent bonds. As the LED methodology evolves to capture increasingly nuanced interaction effects, the complexity of performing these analyses grows proportionally, underscoring the critical need for automated tools.

This paper introduces LEDAW (LED Analysis Wizard), a Python-based program, designed to fully automate LED analysis from input preparation (script-based-only) to output processing with an intuitive GUI. Herein after briefly describing the LED scheme, we provide LEDAW’s design, functionalities, and user workflow, highlighting its key features and advantages.

## 2 Brief Description of LED

### 2.1 Basic LED Terminology

The LED scheme dissects the interaction energy of a supersystem—computed as the difference between the supersystem energy and the sum of subsystem energies at the supersystem geometry—into intra- and interfragment contributions.^32,36^ Here the *supersystem* refers to entire molecular system, composed of two or more chemical entities called “subsystems” (see Figure 1).

**Figure 1:**
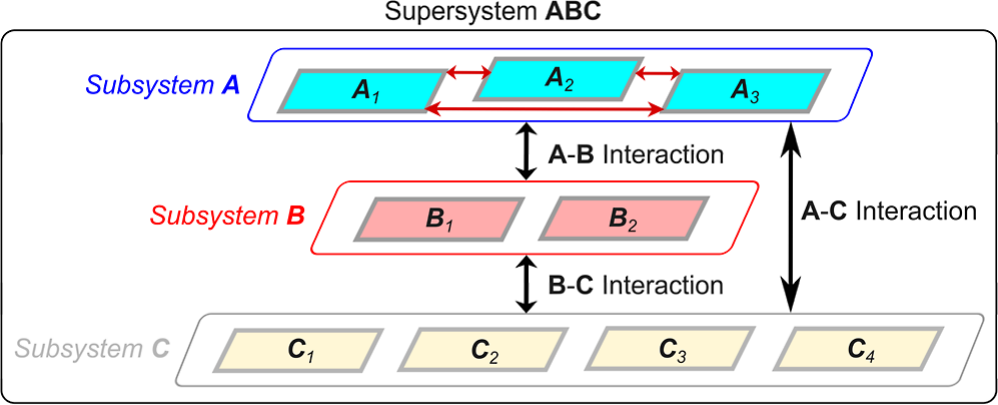
Schematic of a supramolecular complex ABC (*supersystem* or *entire system*), comprising three *subsystems*: A, B, and C. Each subsystem contains multiple *fragments*: A includes *A*_*i*_ (*i* = 1, 2, 3); B includes *B*_*i*_ (*i* = 1, 2); C includes *C*_*i*_ (*i* = 1, 2, 3, 4). Intersubsystem interactions are represented by double-headed black arrows labeled A–B, B–C, and A–C. They are the sum of all fragment-pairwise interactions across the corresponding subsystem pairs, such as, for A–B, all six (3 × 2) interactions between A_1_–B_1_, A_1_–B_2_, A_2_–B_1_, A_2_–B_2_, A_3_–B_1_, and A_3_–B_2_. Intrasubsystem interactions (e.g., A_1_–A_2_) are shown only within A, using double-headed red arrows. LED dissects all these interaction components. Adapted from the LEDAW manual, Copyright 2025, A. Altun.

When investigating interstrand stability of an RNA or DNA triplex, the supersystem is the complete triplex; the three strands are subsystems; and the nucleobases on the strands are fragments. Similarly, in studying interactions between the layers of a supramolecular complex or a molecular crystal, the entire supramolecular assembly is the supersystem; the layers are subsystems; and each individual molecular unit is a fragment. If cluster formation energy of a water hexamer is under consideration, then each water molecule serves as both a fragment and a subsystem. The LED analysis of such interactions, involving a multifragment supersystem, is called as “N-body LED”.

### 2.2 N-Body LED

Let us label fragments as *X* and *Y*, regardless of their subsystem assignment. In standard LED,^13,14^ the total interaction energy, Δ*E*_int_, is decomposed into intrafragment and interfragment contributions as

1 $${\Delta E}_{\mathrm{int}}=\sum_{X}{\Delta E}_{el-prep}^{X}+\sum_{X>Y}\varepsilon^{\left(X,Y\right)}$$

A “geometric deformation energy” (not considered in LEDAW) can be included here to account for the energy required to distort the subsystems from their equilibrium structures to the supersystem geometry.

Here, Δ*E*_el‑prep_^*X*^ is the electronic preparation energy of fragment *X*, arising from the change in its energy as it interacts with all the other fragments. Thus, this term is a cumulative quantity. The fp-LED scheme^36^ decouples this cumulative energy into pairwise Δ*E*_el‑prep_^*XY*^ terms by determining the magnitude of the perturbation of each fragment’s energy by each of the other fragments. This allows rewriting eq 1 as

2 $${\Delta E}_{\mathrm{int}}=\sum_{X>Y}{\Delta E}_{el-prep}^{XY}+\sum_{X>Y}\varepsilon^{\left(X,Y\right)}=\sum_{X>Y}{\Delta E}_{\mathrm{int}}^{XY}$$

here, Δ*E*_el‑prep_^*XY*^ represents perturbation of the energy of fragments *X* and *Y* by each other. While ε^(*X*,*Y*)^ corresponds to the perturbation of the energy of fragment pairs within the same subsystem upon intersubsystems interactions, it corresponds to the genuine intersubsystem interactions between fragment pairs located at different subsystems. This term can be further decomposed into several physically meaningful components

3 $$\varepsilon^{\left(X,Y\right)}=\varepsilon_{elstat}^{\left(X,Y\right)}+\varepsilon_{exch}^{\left(X,Y\right)}+\varepsilon_{no-disp}^{\left(X,Y\right)}+\varepsilon_{disp}^{\left(X,Y\right)}$$

The electrostatic ε_elstat_^(*X*,*Y*)^ and exchange ε_exch_^(*X*,*Y*)^ interaction energies are calculated at the reference level. The nondispersive correlation term ε_no‑disp_^(*X*,*Y*)^ corrects ε_elstat_^(*X*,*Y*)^ to compensate for the characteristic overestimation of dipole moments at the reference level. ε_disp_^(*X*,*Y*)^ is a genuine correlation effect called London dispersion (LD), representing long-range part of the van der Waals potential. If an implicit solvation scheme is used, ε^(*X*,*Y*)^ additionally contains a solute–solvent interaction term ε_solv_^(*X*,*Y*)^.

In the HFLD scheme,^7,8^ both ε_no‑disp_^(*X*,*Y*)^ and correlation part of ΔE_el‑prep_^*X*^ are discarded, while ε_disp_^(*X*,*Y*)^ is obtained in a cost-effective way from DLPNO–CCSD equations. These approximations are well justified for NCIs.^7,8^

### 2.3 Standard and fp-LED Heat Maps

Interaction energy matrices or their heat map representations provide an immediate and intuitive visualization of pairwise interaction strengths. Figure 2 shows N-body “standard LED”^9^ and “fp-LED”^36^ heat maps of the interstrand interaction in a DNA duplex segment, calculated at the DLPNO–CCSD­(T)/def2-TZVP­(-f) level with implicit CPCM water, using the iterative (T_1_) algorithm^5^ and NormalPNO settings with tighter *T*_CutPairs_ = 10^–5^.

**Figure 2:**
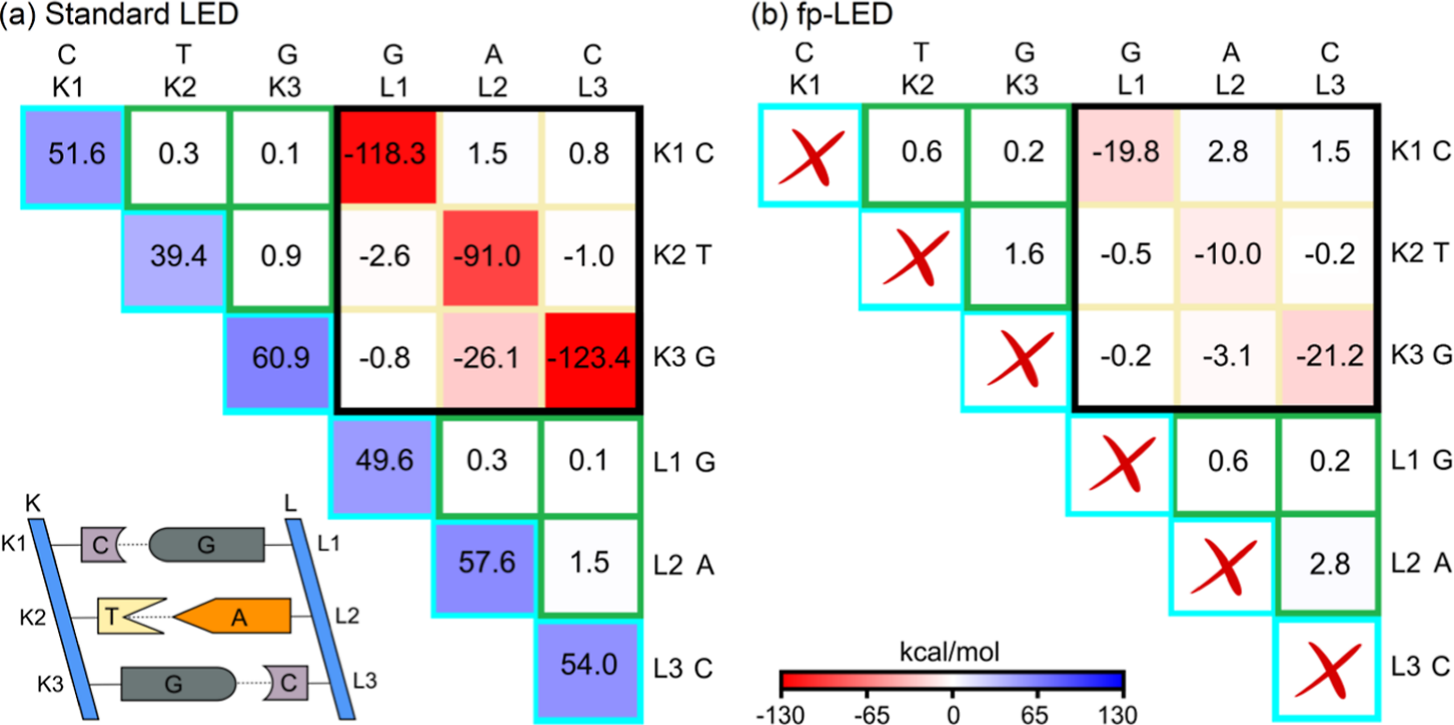
(a) Standard LED and (b) fp-LED heat maps for the BSSE-corrected interaction energy between strands K and L of a DNA duplex segment, with backbones omitted. Subsystems K and L each include three fragments (nucleobases). Heat maps use red for attraction and blue for repulsion. Adapted from the LEDAW manual, Copyright 2025, A. Altun.

In the standard LED heat map (Figure 2a), the diagonal elements enclosed by turquoise lines represent ΔE_el‑prep_^*X*^ terms, each reflecting the cumulative perturbation of a fragment’s energy by all others upon DNA duplex formation.

The fp-LED scheme dissects this cumulative term into fragment-pairwise Δ*E*_el‑prep_^*XY*^ components, explicitly quantifying how much each fragment’s energy is perturbed by every other fragment. Hence, in Figure 2b, each nondiagonal *X–Y* element includes the mutual perturbation between fragments *X* and *Y*.

The nondiagonal ε^(*X*,*Y*)^elements enclosed by green lines represent the perturbation of a given fragment pair’s energy within the same strand upon duplex formation (upper triangle for strand K; lower triangle for strand L). Such differential terms do not exist for single-fragment subsystems.

The submatrix enclosed by a black box represents the genuine ε^(*X*,*Y*)^ interstrand interaction energies of fragment pairs on different strands.

The standard LED heat map (Figure 2a) contains very large positive and negative values, while the fp-LED heat map (Figure 2b) provides values comparable to isolated dimers. Thus, fp-LED directly quantifies interaction strengths between fragment pairs. Although fp-LED values are more intuitive, both approaches yield consistent trends.

The diagonal elements of the submatrix enclosed by the black box are the strongest pairwise energy terms in this DNA example, highlighting base-pairing (hydrogen bonding between nucleobases) as the primary contributor to duplex formation. They also reveal that G-C pairing is about twice as strong as A-T pairing in water. Further decomposition of the nondiagonal terms via eq 3 provides insights into the nature of the chemical interaction, such as electrostatically driven or dispersion-dominated, etc.

### 2.4 Two-Body LED and Cooperativity

Cooperativity refers to the overall influence of surrounding fragments on the interaction energy of a given fragment pair, also called many-body effects. It includes not only classical many-body polarization and electrostatic effects, but also exchange and dispersion components mediated by the environment. Each N-body LED term inherently incorporates such many-body effects. To isolate and quantify them, one can complement the N-body LED analysis with a two-body approximation of the many-body expansion (MBE).^20,36^ This involves performing additional, computationally inexpensive LED calculations on each isolated fragment pair spanning different subsystems.

When LED results for all isolated fragment pairs are assembled into a matrix analogous to the N-body interaction matrix, it forms the two-body LED matrix or heat map. Unlike N-body terms, two-body terms lack environmental contributions, since each pair is treated in isolation.

Subtracting the two-body matrix from the N-body matrix yields the many-body contribution, reflecting cooperative effects from surrounding fragments. Such many-body effects often play a crucial role in molecular crystals, supramolecular assemblies, and biomolecular complexes.^20,36^

Figure 3 shows two-body LED and cooperativity heat maps within fp-LED for the interstrand interaction of the DNA duplex. The cooperativity matrix (Figure 3b) is the difference between the N-body (Figure 2b) and two-body (Figure 3a) matrices. Since the two-body matrix was constructed from isolated fragment pairs on different strands, elements inside green boxes are absent. Hence, corresponding N-body terms are fully cooperative. Environmental effects are significant almost for each interstrand pair, with the strongest on the G-C base-pairing, as seen from diagonal elements of the submatrix enclosed by the black box in Figure 3b.

**Figure 3:**
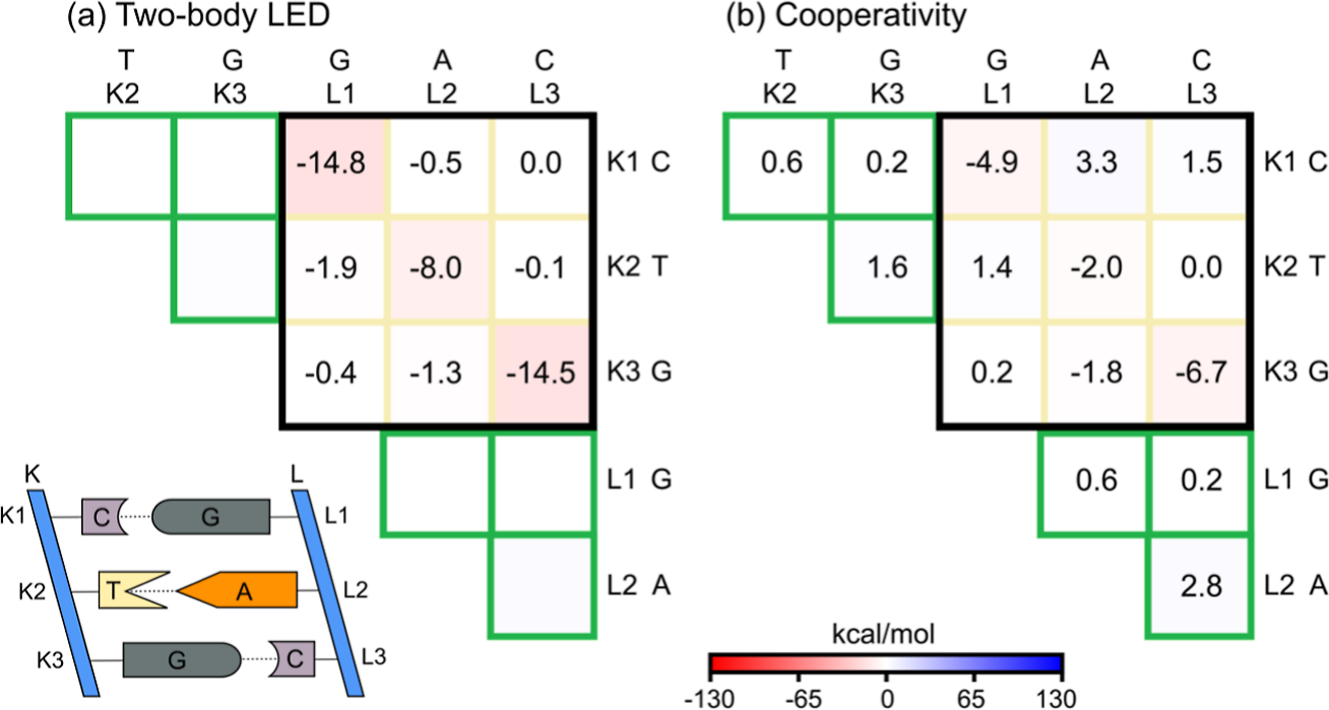
Within fp-LED, (a) two-body LED and (b) cooperativity heat maps for the BSSE-corrected interaction energy between strands K and L of a DNA duplex segment.

### 2.5 Two-Point CBS and CPS Extrapolations

Correlation methods converge slower than mean-field approaches with respect to basis set size. Since large basis set computations are often prohibitively expensive, two-point “Complete Basis Set (CBS)” extrapolations are used to estimate the complete basis limit from energies computed with finite basis sets of successive cardinal numbers (e.g., aug-cc-pVTZ and aug-cc-pVQZ).^43,44^

In DLPNO methods, PNO space truncation significantly enhances computational efficiency. With “TightPNO” cutoffs, DLPNO–CCSD­(T) already achieves 0.5 kcal/mol accuracy on the GMTKN55 superset.^46^ However, for more complex interactions, even tighter settings may be required—limiting applicability. Instead, two-point “Complete PNO Space (CPS)” extrapolation using the energies computed with two *T*_CutPNO_ thresholds of successive exponents (e.g., 10^–6^ and 10^–7^) can be used to obtain results at the complete PNO space limit.^47^ Although DLPNO error scales with system size, CPS extrapolation removes this size dependence, enabling accurate results even for large systems.^48^

CBS and CPS extrapolations account for different effects. Hence, benchmark-quality DLPNO–CCSD­(T) results require both, as illustrated in Figure 4 for the uracil dimer (reaction 17 in the S66 set^49^). Individual LED terms are extrapolated just like the parent reference and correlation energies.

**Figure 4:**
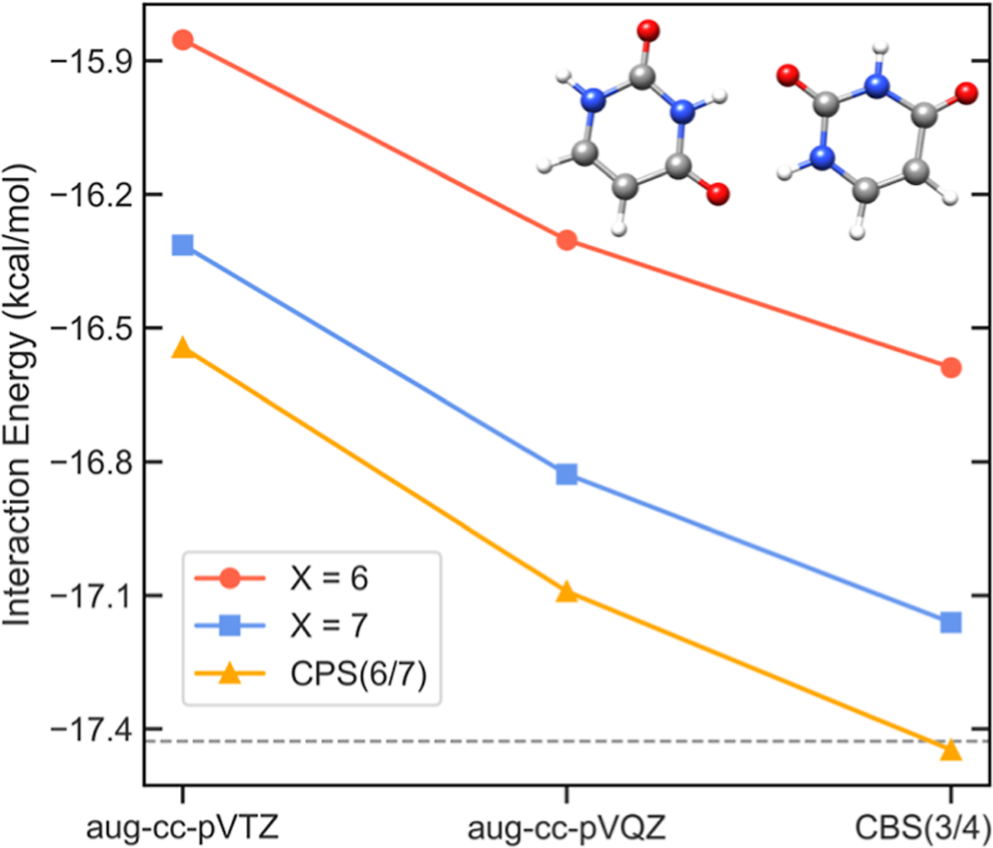
Dependence of the DLPNO–CCSD­(T_1_)/TightPNO interaction energy for the uracil dimer on the *T*_CutPNO_ = 10^–*X*^ threshold and basis set size, along with the corresponding CPS­(6/7) and CBS­(3/4) extrapolated values. The reference horizontal dashed line corresponds to the BSSE-corrected HF/CBS­(3/4) value plus the CCSD­(T) correlation energy calculated with SILVER settings.^50^ Data taken from ref 47.

## 3 Software Architecture

LEDAW consists of two packages: the LED Input Preparator (ledip), which automates the generation of ORCA LED input files; and the LED Analysis Wizard (ledaw), which automates LED analysis using ORCA output files. Hereafter, “LEDAW” (uppercase) refers to the overarching software package or its repository, while “ledaw” (lowercase) denotes its analysis module.

### 3.1 Automatic Input Preparation with Ledip

Preparing LED input files for ORCA is often time-consuming and error-prone, especially for multifragment systems. To illustrate the sheer scale of this task, let us consider a complete LED analysis of interstrand interaction in the three-nucleobase-long DNA duplex KL. For a single computational setting:

N-body LED requires:

- 3 input files: one for the supersystem and one each for subsystems K and L in both BSSE-uncorrected and BSSE-corrected setups.

Two-body LED requires:

- 9 inputs for interstrand fragment pairs (3 × 3),
- 6 inputs for isolated monomers in the BSSE-uncorrected setup (one per fragment),
- 18 inputs for monomers in the BSSE-corrected setup (two per fragment pair, with ghost and real fragments exchanged).

In total, per computational setting:

- 18 inputs for the BSSE-uncorrected setup,
- 30 inputs for the BSSE-corrected setup.

Since CBS and CPS extrapolations each require two computational settings (4 total), a full CBS/CPS-extrapolated N-body and two-body LED analysis demands:

- 72 inputs (18 × 4) in BSSE-uncorrected setups,
- 120 inputs (30 × 4) in BSSE-corrected setups.

These are substantial numbers for a modest six-fragment system—and they grow rapidly with increasing system size. Manual preparation under such conditions is impractical and error-prone.

The ledip package fully automates this process, turning a tedious and error-prone task into a fast and robust workflow. It features two core Python modules, each driven by a dedicated function:

- fragmentation_engine­(): automatically detects and labels molecular fragments from the supersystem’s XYZ file. It offers user-tunable control through three key parameters, enabling precise and flexible fragmentation, even for complex systems:
  - Distance cutoff to set the maximum bonding distance
  - Coordination number to handle coordination bonds
  - Custom bond list to assign specific bonds to separate fragments
- led_input_prep_engine­(): takes an XYZ file with fragment labels and the corresponding fragment groupings for each subsystem, then generates—within seconds—all required ORCA LED input files for both N-body and two-body calculations, including both BSSE-corrected and BSSE-uncorrected setups.

### 3.2 Automating LED Analysis with Ledaw

As discussed above, a complete LED analysis may involve hundreds of separate ORCA calculations, even for modest systems. Moreover, each calculation can yield hundreds of decomposed interaction terms—especially in multifragment supersystems—making manual postprocessing laborious and error-prone.

The ledaw package automates the entire postprocessing workflow, typically completing it within a minute, thus dramatically reducing manual effort and turnaround time. It calculates each N-body LED, two-body LED, and cooperativity interaction term from ORCA output files using both standard and fp-LED, compiles the results into interaction energy matrices, and generates heat maps. It also supports CBS and CPS extrapolations of LED terms, crucial for achieving benchmark accuracy. Beyond basic supramolecular interaction energy component calculations, ledaw enables fragment-pairwise decomposition of electronic preparation and solute–solvent interactions, and computes triples dispersion (see ref 36 and the user manual).

To minimize manual effort, ledaw automatically parses ORCA output files, including fragment coordinates, labels, and real vs ghost identities. Users need only specify the output file paths and a few optional parameters for customization. Complex tasks, such as excluding redundant correlation terms in HFLD (or corresponding multilevel DLPNO–CCSD) outputs, mapping BSSE-uncorrected and BSSE-corrected subsystems to supersystem or two-body files, and managing implicit solvation terms, are all handled internally, ensuring a streamlined and user-friendly workflow.

Ledaw includes five core engine functions: engine_LED_N_body­(), engine_LED_two_body­(), cooperativity_engine­(), extrapolate_engine­(), and heatmap_plot_engine­(). Users can interact with ledaw via both script-based and GUI-based workflows. The script-based approach is ideal for code-oriented users or for integrating ledaw into customized analysis pipelines. The LEDAW repository provides well-annotated example scripts, which demonstrate the use of these functions and can themselves be easily adapted to other systems by simply updating file and directory paths.

The LEDAW-GUI, developed using PySide6 (Qt for Python), frees users from typing commands and file paths—tasks prone to errors. With just a few clicks and file selections, the GUI simplifies the ledaw workflow. It also features helpful information boxes that guide users step-by-step, offering in-place assistance without the need to consult the detailed user manual.

Figure 5 shows the Home tab of the LEDAW-GUI. It lets users select LED analysis using the DLPNO–CCSD­(T), DLPNO–CCSD, or HFLD method. When CBS is selected, the GUI offers three options: CBS­(2/3), CBS­(3/4), and a custom parameter field. The default CBS­(2/3) and CBS­(3/4) parameters are optimized for the (aug)-cc-pV*n*Z family.^45^

**Figure 5:**
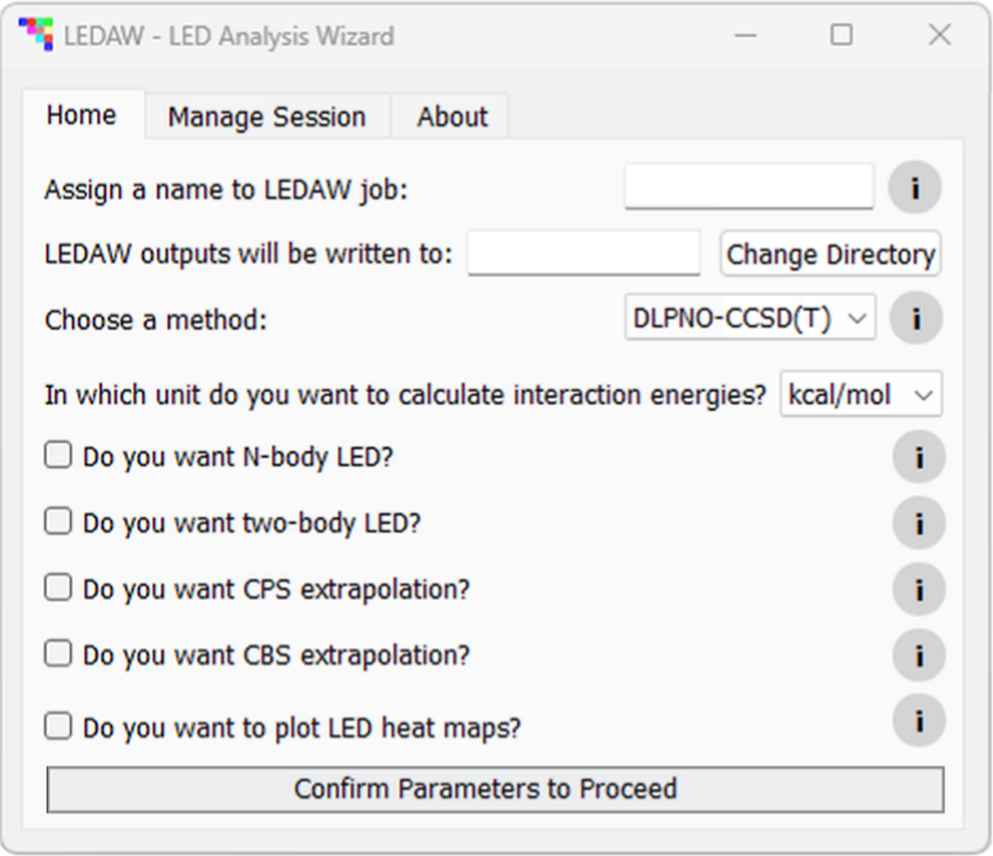
Home tab of the LEDAW-GUI, controlling the generation and content of subsequent tabs based on user selections.

Based on the selections made in this tab, additional tabs—N-body, Two-body, Plot, and Run—are dynamically generated, with contents adapting accordingly. The N-body and Two-body tabs allow selecting ORCA output files necessary for these analyses. The N-body tab also permits specifying alternative N-body output files. This addresses a common challenge in large-scale quantum chemistry calculations, where jobs may crash after completing certain parts (e.g., HF/LED but not correlation decomposition) due to memory or time limitations. In such cases, users can disable HF/LED decomposition in the follow-up run for efficiency. If an alternative output file is provided, ledaw automatically attempts to retrieve missing data from it when absent in the main file. This greatly enhances workflow resilience by salvaging partial results from incomplete ORCA runs and avoiding costly full resubmissions.

By default, during the N-body LED analysis, fragment labels in subsystem files are aligned with those in the main supersystem output. The N-body tab also allows customizing these fragment labels. When both N-body and two-body LED analyses are requested, two-body LED inherits fragment labels from the N-body LED to ensure consistency. Otherwise, two-body fragment labels are assigned based on the order of the monomer output file list. The Plot tab offers options for designing the heat maps, while the Run tab displays the ledaw run status.

If both N-body and two-body LED analyses are requested, the GUI automatically performs a cooperativity analysis by calling the cooperativity_engine­(), which subtracts two-body LED matrices from the N-body matrices. For two-fragment systems, the N-body and two-body modules are functionally identical, enabling cooperativity analysis to serve as a tool for evaluating differential effects under varying computational settings. For example, when BSSE-corrected and BSSE-uncorrected LED outputs are assigned to N-body and two-body tabs (or vice versa), cooperativity analysis reflects the BSSE correction. Basis set effects can be evaluated similarly. For multifragment systems, such comparisons can be run outside the GUI via calling engine_LED_N_body­() twice and cooperativity_engine­() once.

At the end of a ledaw run, Excel files containing all LED interaction energy (sub)­matrices within both standard and fp-LED are generated for all selections made in the Home tab. If selected, corresponding heat maps are also produced as figure files.

## 4 Conclusions

This paper introduces LEDAW, a Python-based software that automates the complex and error-prone processes of (i) generating ORCA LED input files through the ledip package, and (ii) postprocessing ORCA output files to generate LED interaction energy matrices and heat maps through the ledaw package. LEDAW allows completing entire preprocessing and postprocessing pipelines in minutes, a task that would otherwise take several hours, if not several days.

LEDAW is a powerful toolkit for analyzing complex molecular interactions from ORCA output files, offering an intuitive GUI, flexible script-based workflows, and comprehensive analysis methods. These include N-body, two-body, and cooperativity analyses within both standard and fp-LED, alongside CBS and CPS extrapolations. LEDAW’s customizable heat maps facilitate effective communication and dissemination of scientific results.

## Data Availability

The LEDAW software—including its source code, comprehensive user manual (covering theoretical background, tips for setting ORCA input files, functionalities, and usage), example files for preprocessing, sample ORCA LED output files for various interaction types (including the DNA example reported here), and Python scripts for their analysis as an alternative to the GUI usage—is available at https://github.com/ahmetaltunfatih/LEDAW.
